# Genetic manipulation of anti-nutritional factors in major crops for a sustainable diet in future

**DOI:** 10.3389/fpls.2022.1070398

**Published:** 2023-02-15

**Authors:** Aishwarya Duraiswamy, Nancy Mano Sneha A., Sherina Jebakani K., Sellakumar Selvaraj, Lydia Pramitha J., Ramchander Selvaraj, Indira Petchiammal K., Sharmili Kather Sheriff, Jenita Thinakaran, Samundeswari Rathinamoorthy, Ramesh Kumar P.

**Affiliations:** ^1^ Genetics and Plant Breeding, School of Agricultural Sciences, Karunya Institute of Technology and Sciences, Coimbatore, India; ^2^ Agronomy, School of Agricultural Sciences, Karunya Institute of Technology and Sciences, Coimbatore, India; ^3^ Horticulture, School of Agricultural Sciences, Karunya Institute of Technology and Sciences, Coimbatore, India; ^4^ Crop Physiology, School of Agricultural Sciences, Karunya Institute of Technology and Sciences, Coimbatore, India; ^5^ Plant Biochemistry, School of Agricultural Sciences, Karunya Institute of Technology and Sciences, Coimbatore, India

**Keywords:** anti-nutritional factors, regulatory pathways, plant breeding, food processing, gene editing

## Abstract

The consumption of healthy food, in order to strengthen the immune system, is now a major focus of people worldwide and is essential to tackle the emerging pandemic concerns. Moreover, research in this area paves the way for diversification of human diets by incorporating underutilized crops which are highly nutritious and climate-resilient in nature. However, although the consumption of healthy foods increases nutritional uptake, the bioavailability of nutrients and their absorption from foods also play an essential role in curbing malnutrition in developing countries. This has led to a focus on anti-nutrients that interfere with the digestion and absorption of nutrients and proteins from foods. Anti-nutritional factors in crops, such as phytic acid, gossypol, goitrogens, glucosinolates, lectins, oxalic acid, saponins, raffinose, tannins, enzyme inhibitors, alkaloids, β-*N*-oxalyl amino alanine (BOAA), and hydrogen cyanide (HCN), are synthesized in crop metabolic pathways and are interconnected with other essential growth regulation factors. Hence, breeding with the aim of completely eliminating anti-nutrition factors tends to compromise desirable features such as yield and seed size. However, advanced techniques, such as integrated multi-omics, RNAi, gene editing, and genomics-assisted breeding, aim to breed crops in which negative traits are minimized and to provide new strategies to handle these traits in crop improvement programs. There is also a need to emphasize individual crop-based approaches in upcoming research programs to achieve smart foods with minimum constraints in future. This review focuses on progress in molecular breeding and prospects for additional approaches to improve nutrient bioavailability in major crops.

## Introduction

Consumption of foods for a sustainable diet has the potential to reduce hidden hunger in many countries. One of the major factors influencing nutrient absorption is the presence of anti-nutrients in foods (Thakur et al. 2019). These have largely been overlooked by research projects that aim to minimize nutritional deficiencies and toxicities in diets in the growing population (Gilani et al., 2012). Anti-nutritional factors in foods hinder digestion and reduce the bioavailability of the major nutrients. In some severe cases, they are a major contributor to serious disorders and, when intake is excessive, can even cause death (Frick et al., 2017). Hence, this has to be rectified in major food crops so that the mineral uptake from plant-based foods is unaltered. The major anti-nutritional factors in foods include phytic acid, raffinose, saponins, tannins, enzyme inhibitors, lectins, gossypol, glucosinolates, goitrogens, oxalic acid, erucic acid, alkaloids, β-*N*-oxalyl amino alanine (BOAA), and hydrogen cyanide (HCN) (Thakur et al., 2019; Samtiya et al., 2020). These factors play a major role in human health, as they hinder nutrient absorption and uptake *via* chelation and enzyme inhibition. Legumes are of particular concern, as they contain a comparatively higher proportion of anti-nutritional traits than other crops (Parca et al., 2018). This presumes that consumer favour less consumption of these crops despite their potential nutritive traits (Jaiswal, 2020).

Several traditional processing techniques, such as soaking, roasting, sprouting, fermentation, boiling, and extrusion, can reduce anti-nutritional components in grains. However, these techniques are adopted at a small scale in household cooking and in value-added products from agro-industries (Das et al., 2022). Industrial organizations utilize these processing methods to enhance the bioavailability of food grains in processed foods. Eliminating anti-nutrients in foods remains a major objective, and one that could be achieved by using advanced techniques, such as RNAi and gene editing, to develop high-nutrition crops. The reduction of anti-nutritional traits has been a progressively intense area of research since the 1950s, but there are several barriers to improving varieties by reducing anti-nutritional factors. The accumulation of anti-nutrients in crops is still to be completely explored for all the major traits (Tong et al., 2021). Some anti-nutrients have been explored more than others, and the genes responsible for their biosynthesis offer a major way of altering the concentrations of anti-nutrients in foods. Phytic acid, raffinose, glucosinolates, enzyme inhibitors, and erucic acid are the anti-nutrients that have been the predominant focus of breeding and transgenic approaches. Saponins, oxalic acid, alkaloids, HCN, goitrogens, and BOAA need to be further studied in the future (Thakur et al. 2019).

Another major factor in reducing these antinutrients in crops is their stable expression across locations. Anti-nutrients such as phytic acid, glucosinolates, and alkaloids are highly influenced by soil, fertilizer applications, and other edaphic factors (Zhuo et al., 2013; Frick et al., 2017; Pramitha et al., 2021). Therefore, alternate strategies involving advanced multi-omics accompanied by rapid estimation techniques and gene editing protocols play an essential role in optimizing the nutrient availability of major crops and developing non-toxic foods for human consumption. However, it is also important to monitor the effects of reduction of anti-nutrients in crops, as anti-nutrients such as saponins, raffinose, enzyme inhibitors, gossypol, glucosinolates, and phytic acid have a major role in plant growth metabolism (Sahu et al., 2020). Previous reports have shown that these compounds constitute a regulation on crop metabolism and growth (Rodríguez-Sifuentes et al., 2020; Pramitha et al., 2021; Elango et al., 2022). Thus, a focus on the reduction of negative pleiotropic effects on characteristics such as seed quality, seed yield, and stable expression, and on the influence of edaphic factors on nutrient accumulation, processing, and storage, are necessary to develop a high-value food crop with mineral availability in the near future (Coulibaly et al., 2011). Among all the major crops, soybean is the one that has been most explored for reducing anti-nutrients, followed by brassicas and cotton, which have been investigated to improve their overall acceptance for human and animal feed (Rathore et al., 2020; Le et al., 2020). Hence, this review highlights progress in research into breeding for anti-nutritional traits in major food crops and also predicts its future direction.

## Major anti-nutritional traits in food crops and their effects on consumption

There are several anti-nutritional factors in cereal- and legume-based foods, and some of the major key anti-nutritional traits are elaborated here. The major factors that interrupt food digestion and absorption are phytic acids, gossypols, lectins, raffinose, enzyme inhibitors, goitrogens, saponins, tannins, oxalic acid, erucic acid, alkaloids, BOAA, and HCN. This section describes the effects of consumption of these anti-nutrients in foods and specifies levels of consumption in regular diets (**Table 1**).

**Table 1:** The major role of anti-nutrients in consumption and plant growth regulation.

S. no.	Anti-nutrient	Effects on consumption	Role in plant growth	Pathway	Reference
1.	Phytic acid	i. Nutritional inhibitor in monogastric animals ii. Decreases the risk of colon cancer and inflammatory bowel disease iii. Lowers blood glucose level	i. Phosphorus storage and chelation of micronutrients for growth and development	Myoinositol pathway	Gupta et al., 2015; Dilworth et al., 2005; Graf and Eaton, 1990
2.	Raffinose	i. Raffinose not digested by humans and monogastric animals ii. Leads to flatulence in humans and animals iii. Prevents non-alcoholic fatty liver disease in humans iv. Reduces inflammation, diabetes, allergies, and obesity	i. Acts as a cryoprotectant ii. Acts as a storage metabolite and is absorbed in seeds and roots iii. Acts as a source of energy for seed germination	Inositol phosphate pathway	Kannan et al., 2018; Elango et al., 2022
3.	Gossypol	i. Acute poisoning on ingestion ii. Causes iron deficiency known as erythropoiesis iii. Increases cytosolic Ca^2+^ activity iv. Decreases antioxidant levels in tissues	i. Resistance to cotton bollworm	Sesquiterpenoid aldehyde pathway	Soto-Blanco, 2008; Gadelha et al., 2011; Randel et al., 1996; Zhang et al., 2007; Mena et al., 2004; Zbidah et al., 2012; Bottger et al., 1964; Kovaci, 2003
4.	Saponins	i. Cause diarrhea and vomiting by damaging red blood cells ii. Affects the nutrient absorption by gut membranes iii. Negative impact on chick development and feed efficiency	i. Act as phytoalexin during fruit and tuber development ii. Resistance against diseases in vegetables	Cytosolic mevalonic acid pathway	Akande et al., 2010; Ribera and Zuñiga, 2012; Cárdenas et al., 2015
5.	Goitrogen	i. Deficiency of thyroid hormone ii. Reduces growth and reproductive performance iii. Apoptotic and anti-proliferative effects in thyroid cancer cells	–	Glycosyl transferase pathway	Akande et al., 2010; Chatterjee et al., 2018; Boncompagni et al., 2018
6.	Glucosinolates	i. Cause rancidity ii. Prevent cardiovascular and neurodegenerative diseases	–	Aliphatic glucosinolate pathway	Kamal et al., 2022
7.	Oxalic acid	i. Causes headache, coma, and kidney stones ii. Calcium oxalate has a severe impact on human nutrition and health iii. Leads to death due to oxalate poisoning	i. Precursors of oxalic acid play a major role in climate resilience ii. Growth regulation of crops during pollination	–	Egbuna, 2018; Awulachew, 2022
8.	Erucic acid	i. fat accumulation in heart muscles ii. cardiovascular diseases and myocardial lesions in the heart	–	–	Wani et al., 2022
9.	Lectin	i. Agglutinates red blood cells ii. Anti-tumor agent iii. Antimicrobial, antifungal, antibacterial, antiviral iv. Alters the integrity of intestinal mucosa	i. Regulation of cell signaling and plant response to biotic, abiotic, and symbiotic stimuli	–	López-Moreno et al., 2022
10.	Enzyme inhibitors	i. Trypsin inhibitors trigger pancreatic hyperplasia ii. Prevention of type 2 diabetes and obesity iii. Protease inhibitors reduce the activity of proteolytic enzymes during ingestion iv. Alpha-amylase inhibitors affect post-meal plasma glucose levels	Confer biotic stress tolerance and act as biopesticides	–	Bhutkar and Bhise, 2012; Battelino et al., 2019; do Amaral et al., 2022; Ribeiro et al. 2015
11.	Tannins	i. Inhibit digestive enzymes and cause intestinal damage ii. Have been associated with reduced feed intake, growth rate, feed efficiency, and protein digestibility iii. Enhance the food product’s oxidative stability iv. Improve the quality of the meat and milk. Act as a natural preservative	i. Antiparasitic properties of plant tannins ii. Act against pathogenic bacteria, have antibacterial actions, and are antioxidants iii. Prevent neurodegenerative diseases and have anti-tumor, anti-inflammatory, and antibacterial properties	Shikimate pathway	Akande et al., 2010; Gemede and Ratta, 2014; Gilani et al., 2012 Tong et al., 2021; Mora et al., 2022
12.	HCN	i. In animals stops cellular respiration process due to asphyxia ii. Severe shortness of breath and frequent urination in animals	–	–	Al-Beiruty et al., 2020
13.	BOAA	i. Causes neurolathyrism, a neurologic condition that is irreversible in both humans and animals	Act as an Antioxidant	Begins with the formation of BIA from *O*-acetyl- L-serine (OAS)	Das et al., 2021

### Phytic acid

Phytic acid (C_6_H_18_O_24_P_6_) is a naturally occurring antioxidant that chelates positively charged minerals such as phosphorus, iron, and zinc (Raboy et al., 2000). It is found primarily in the grains, nuts, and seeds of cereals, legumes, and vegetables. Phytic acid is found in rice aleurone, and it is also abundant in the endosperm and embryo of maize (Raboy et al., 2000). Phosphorus is primarily stored in the form of phytic acid in seeds after pollination. During germination, it is degraded by the enzyme phytase to support plant growth and development (Pramitha et al., 2021). Monogastric animals lack the enzyme phytase in their digestive tract, and as a result phytic acid acts as a nutritional inhibitor by chelating the available micronutrients in foods (Gupta et al., 2015). The non-dissolvable form of phytic acid, i.e., the mineral-bound complex, and remains a problem, as its excretion in animal feces results in eutrophication and soil pollution (Raboy et al., 2001). Hence, reducing phytic acid in grains is a beneficial solution to enhance mineral availability following consumption (Pramitha et al., 2021). Despite these anti-nutritional features, dietary phytic acid has been found to reduce the risk of colon cancer and other inflammatory bowel diseases by acting as a beneficial antioxidant in foods. Its inclusion in foods thereby prevents lipid peroxidation, oxidative spoilage, discoloration, putrefaction, and syneresis. Hence, the reduction of phytic acid in foods should be optimized for normal growth and regulation of metabolism. The safest range for overall phytic acid consumption is reported to be around 250–800 mg (Graf and Eaton, 1990).

### Gossypol

Gossypol (C_30_H_30_O_8_) is a group of polyphenols that can cause acute poisoning on ingestion (Stipanovic et al., 1975). Studies of gossypol report that cumulative toxic effects can occur after just 1–3 months of consumption (Soto-Blanco, 2008; Gadelha et al., 2011). It is safest to limit gossypol consumption to 20 mg of gossypol per kg of feed. Poisoning by gossypol has been reported in broiler chicks, pigs, dogs, sheep, and goats. However, gossypol toxicity is more severe in monogastric animals such as pigs, birds, fish, and rodents than in ruminants (Kenar, 2006; Alexander et al., 2008). The effect of gossypols is more severe in younger ruminants than in adults. The major impact of ingestion is anemia, which is frequently observed in cottonseed-fed animals. During ingestion, gossypol binds with iron in hemoglobin to form a gossypol–iron complex, which inhibits iron absorption, resulting in a deficiency known as erythropoiesis, i.e., erythrocyte fragility (apoptosis-like erythrocyte death) (Randel et al., 1996; Mena et al., 2004; Zhang et al., 2007). Further, this increases cytosolic Ca^2+^ activity, which causes cell membrane scrambling and contraction (Zbidah et al., 2012). In addition, clinical signs of gossypol poisoning are linked to decreased antioxidant levels in tissues (Kovaci, 2003). Hence, gossypol reduces energy generation from oxidative metabolism at high concentrations by interfering with enzymatic activity in the mitochondrial electron transport chain and oxidative phosphorylation. In addition, gossypol has an impact on both male and female gametogenesis and promotes embryo lesions linked to male infertility (Gadelha et al., 2011). Therefore, gossypol could be explored for its potential use as a male contraceptive in future pharmaceutical research (Soto-Blanco, 2008; Chang et al., 2011).

### Lectins

Lectins (complex carbohydrate-binding proteins) are a type of glycoprotein with non-catalytic carbohydrate-binding sites that are classified into animal, algal, bacterial, fungal, and plant lectins (Mishra et al., 2019). Lectins are also known as hemagglutinins. These “anti-nutrients” have received a lot of attention because of their role in obesity, chronic inflammation, and autoimmune diseases. They are predominantly observed in raw legumes such as kidney beans, lentils, peas, soybeans, and peanuts, and in whole grains such as wheat. In leguminous plants, lectin content is higher in seeds than in bark, leaves, roots, or stem. Plant lectins are generally found in nuts, cereals, and leguminous seeds (El-Araby et al., 2020). Consumption of lectins in their active state, for example the consumption of even small amounts of raw or undercooked kidney beans, can cause severe adverse reactions in humans. Kidney beans contain phytohemagglutinin, a lectin that causes red blood cells to aggregate, leading to cause nausea, vomiting, stomach upset and diarrhea (Peumans and Van Damme, 1995). Bloating and flatulence are milder side effects. Active lectins have been found in animal cell studies to interfere with mineral absorption, affecting the concentrations of calcium, iron, phosphorus, and zinc in the digestive tract (Vasconcelos and Oliveira, 2004). Thus, 200–400 hemagglutinin units (hau) is considered a safe level for consumption of lectins from leguminous foods (Van Damme et al., 2008; Kobayashi et al., 2014). Despite their negative side effects, lectins have been shown to be useful for cancer treatment due to their antiangiogenic, antimetastatic, and antiproliferative activity (Bhutia et al., 2016; Panda et al., 2018; Sinha et al., 2019).

### Raffinose

Pulses are rich in carbohydrates, proteins, dietary fiber, vitamins, minerals, and other bioactive substances in the human diet. However, their consumption and acceptance are constrained globally, particularly in industrialized countries, due to the high proportion of raffinose family oligosaccharides (RFOs). These are found in beans, cabbage, Brussels sprouts, broccoli, asparagus, and whole grains (Elango et al., 2022). RFOs (C_18_H_32_O_16_) is prevalent in the seeds of legume families such as chickpea (*Cicer arietinum*), lentil (*Lens culinaris*), and soybean (*Glycine max*). They are also found in the leaves and tubers of vegetables and in other specialized storage organs such as roots. Raffinoses are found in the tubers of Chinese artichoke (*Stachys sieboldii*) and in the leaves of a common bugle (*Ajuga reptans*). Defatted soy flour has an average range of raffinose from 1.15%-3.23% espectively. In lentil, RFOs level ranges from 4.5 to 5.5 mol 100 g^–1^ of flour, and in faba bean it ranges from 0.12% to 0.29% (Johnson et al., 2021).

Humans and monogastric animals cannot digest RFOs; instead they are fermented by the microflora of the large intestine. This fermentation produces carbon dioxide, hydrogen, and methane, causing flatulence and stomach discomfort (Kannan et al., 2018). However, RFOs also confer beneficial effects, such as antiallergic, anti-obesity, and anti-diabetic effects, the prevention of non-alcoholic fatty liver disease, and cryoprotection. They positively affect the gut microbiota and the health of the large intestine. Hence, RFOs could be used as therapeutic agents to reduce inflammation, diabetics, and allergies. As RFOs are considered the main cause of flatulence in humans and animals, there is a need to strike the right balance of RFOs content in crops if they are to be promoted as functional foods (Elango et al., 2022).

### Enzyme inhibitors

Protease inhibitors are naturally occurring plant inhibitors that have become a focus of research due to their effective method of limiting enzyme activity through protein–protein interactions. They inhibit enzyme activity *via* the catalytic mode by blocking the enzymes’ active sites. Cereals contain substantially less of these digestive inhibitors than legumes (Nikmaram et al., 2017). Protease inhibitors substantially reduce the activity of proteolytic enzymes during ingestion (Troll and Wiesner, 1983). There are various enzyme inhibitors, among which trypsin inhibitors and alpha-amylase inhibitors are the major enzyme inhibitors in foods. Alpha-amylase primarily influences carbohydrates, namely polysaccharides, which are broken down to form oligosaccharides. Therefore, enzyme inhibitors that inhibit alpha-amylase activity will boost carbohydrate levels by slowing the digestion of carbohydrates, having an impact on the typical post-meal levels of plasma glucose (Bhutkar and Bhise, 2012). Speaking of the Trypsin inhibitors also enhance the production of hormones such as steatogenic hormone and cholecystokinin (CCK) and this would reduce food intake and body weight (Cristina Oliveira de Lima et al., 2019). In humans, consumption of trypsin inhibitors can reduce growth rate, slow protein digestion, and reduce amino acid availability, triggering pancreatic hyperplasia (Adeyemo and Onilude, 2013). Several studies have found that the inhibition of some enzymes, namely alpha-amylase, alpha-glucosidase, and lipase, is beneficial, increasing the digestibility of legume-based foods. Although it has health advantages associated with the prevention of type 2 diabetes and obesity, malfunctions relating to digestion have to be overlooked in the future (Li and Tsao, 2019).

### Goitrogens

Goitrogens (C_5_H_7_NOS) got their name from “goiter,” which means “abnormal growth”. Goiter is the enlargement of the thyroid gland due to a deficiency of thyroid hormone. Soybean and cassava are cruciate vegetables of the genus *Brassica* and are rich in goitrogens. However, high goitrogen concentrations have also been reported in other cruciferous vegetables (Truong et al., 2010). Goitrogens interfere with iodine utilization and with thyroid hormone production. Deficiency of thyroid hormone thus results in reduced growth and reproductive performance of an individual. The effect of goitrogens can be reduced by iodine supplementation than by heat treatment (Akande et al., 2010). Foods containing goitrogens also contain different bioactive compounds that protect against thyroid cancer (Fiore et al., 2020). Crucifers contain sulforaphane, an isothiocyanate that has been observed to possess an apoptotic and antiproliferative effect in thyroid cancer cells (Chatterjee et al., 2018). Goitrogens have also been used in the treatment of COVID-19 to activate *Nrf2-Keap1* and counteract the COVID-19-induced cytokine storm (Bousquet et al., 2021; Singh et al., 2021). Hence, safe consumption of these compounds needs to be ensured to avoid their negative side effects.

### Saponins

Saponins (C_58_H_94_O_27_) are non-volatile, surface-active secondary metabolites found in soybeans, sugar beets, peanuts, spinach, asparagus, broccoli, potatoes, apples, eggplants, alfalfa, and ginseng root. Saponins are glycosidic triterpenoids that are widely distributed in the seed coat of crops (Faizal and Geelen, 2013). They are structurally diverse and chemically are known as triterpenes and steroid glycosides (Khodakov et al., 1996). The structural complexity of saponins is responsible for their varied physical, chemical, and biological properties, including sweetness, bitterness, and foaming and emulsifying properties. Hence, saponins have pharmacological, medicinal, hemolytic, antimicrobial, insecticidal, and molluscicidal activities (Sparg et al., 2004). Consumption of saponins often cause diarrhea and vomiting and also leads to the breakdown of red blood cells. It has also been demonstrated that saponins can attach to intestinal cells and influence nutrient absorption in gut membranes. Furthermore, it has been noted in the poultry sector that saponins have a negative impact on chicks’ development, feed efficiency, and ability to absorb dietary lipids, cholesterol, bile acids, and vitamins A and E (Akande et al., 2010).

### Tannins

Tannins (C_76_H_52_O_46_) are plant polyphenolic compounds that bind to and precipitate proteins and other organic compounds such as amino acids and alkaloids. They combine with vitamin B_12_ to produce complexes during digestion. Hydrolyzable tannins and proanthocyanidins (PAs) are the two types of tannins (condensed tannins). Hydrolyzable tannins are more resistant to enzymatic and non-enzymatic hydrolysis than PAs, which are usually more water soluble (Chukwuebuka and Chinenye, 2015). Condensed tannins are abundant in leguminous forages and seeds. Thus, tannins combine with dietary proteins to form a digestible complex that binds to and thus inhibits endogenous proteins, including digestive enzymes (Moses et al., 2022). In addition, they have anti-nutritional effects that can lead to intestinal damage and interfere with iron absorption, and they can be carcinogenic (Akande et al., 2010). As tannic acid it is also used in the manufacture of rubber, inks, and dye fixatives. For consumption, reduction of tannins in foods leads to a healthier digestive tract.

### Oxalic acid

Oxalic acid (C_2_H_2_O_4_) is the dicarboxylic acid that appears as a potassium and calcium salt in the cell sap of *Oxalis* and *Rumex* species of plants. After passing through the digestive system, insoluble compounds of oxalic acid (calcium oxalate) cannot be excreted *via* the urinary tract. This can result in kidney stones, and thus calcium oxalate can have a severe impact on human nutrition and health. Cruciferous vegetables such as kale, radishes, cauliflower, and broccoli, as well as chard, spinach, parsley, beets, black pepper, chocolate, nuts, berries, and beans, are rich in oxalates (Awulachew, 2022). Calcium supplements are suggested to be consumed with foods high in oxalic acid to expel calcium oxalate from the gut and reduce the levels of oxalates in blood. Although rare, consumption of oxalates can cause kidney disease or even death due to oxalate poisoning (Chukwuebuka and Chinenye, 2015).

### Erucic acid

When triglycerides containing erucic acid in the lipids are digested, erucic acid is released into the bloodstream and distributed to tissues for release of energy through oxidation from mitochondrial cells in muscles. However, erucic acid oxidation in cardiac muscles are low. Thus, this results in the accumulation of fat in heart muscles, which causes cardiovascular diseases and myocardial lesions in the heart (Wani et al.2022).

### Alkaloids

Alkaloids, especially quinolizidine, found in commercial legumes such as lupins (C_10_H_19_NO), are highly toxic when consumed. These secondary metabolites are specific to the genera *Lupinus*, *Baptisia*, *Thermopsis*, *Genista*, *Cytisus*, *Echinosophora*, and *Sophora* of the Leguminosae family. Consumption of these alkaloids at a high concentration leads to acute anticholinergic toxicity, the symptoms of which include blurry vision, headache, weakness, and nausea (Frick et al., 2017). It has also been also observed that the dose range of 11–25 mg/kg is lethal to children. However, so far, no fatalities in adults have been recorded (Daverio et al. 2014). Although *Lupinus* is a genus that has been domesticated only recently, four species containing toxic quinolizidine alkaloids (QAs) are cultivated. This is a major concern, and the threshold level of consumption considered safe is 0.02% alkaloid. Studies on QAs have been initiated and more should be carried out in the upcoming years. To date, only a few studies of alkaloids such as nicotine, vinblastine, vincristine, berberine, and morphine in economically important crops have been conducted (Frick et al., 2017).

## Other anti-nutrients with health effects

Hydrogen cyanide (HCN) is a toxic chemical whose consumption has adverse effects in animals and humans. This is a major issue in fodder sorghum and sorghum during the earlier vegetative growth. Techniques to enable rapid detection of low HCN levels are being developed, and the latest advancements enable breeding of low-HCN types of sorghum (Fox et al., 2012; Al-Beiruty et al., 2020).

BOAA is a neurotoxin in seeds and leaves. BOAA is a by-product of nitrogen metabolism in plants and is a major problem in *Lathyrus sativus*, consumption of which causes a non-reversible neurologic disorder known as lathyrism. Although wide variations in the germplasm have been reported, further studies on the nature and actions of genes involved in BOAA biosynthesis are needed. Few molecular breeding techniques along with omic approach, intron based markers and gene editing are being standardized for reducing BOAA content in *Lathyrus*, as this is a major rice fallow crop in South Asian countries (Tripathy et al., 2015; Das et al., 2021). Varieties such as Pusa-24, Pusa-305, LSD-1, LSD-2, and LSD-3 are lower BOAA cultivars containing less than 0.2% BOAA (Gupta et al., 2021).

## Regulatory role of anti-nutritional factors in crops and their biosynthesis

Anti-nutritional traits are compounds that interfere with the bioavailability of nutrients. They also serve as an integral part of growth and metabolism in plants. Hence, understanding their metabolism exhibits their role in regulation and facilitates genetic manipulation. The identification of anti-nutritional traits in crops, and of their wide range of pleiotropic effects, would provide a further basis for alternate strategies to overcome their constraining effects for developing high-nutritional crops (**Table 1**).

### Phytic acid

Phytic acid is one of the most ubiquitous anti-nutritional factors, being present in the aleurone layer of cereals, maize embryo, and the cotyledon of legumes. It is synthesized by the myoinositol pathway, which is a part of starch and glucose metabolism in cells. The pathway is of two types: a lipid-independent pathway is found in seeds and a lipid-dependent pathway occurs in leaves. The lipid-independent pathway comprises the sequential phosphorylation of the six-carbon cyclic alcohol *myo*inositol (Ins) and soluble inositol phosphates (InsPs). However, the lipid-dependent pathway uses phosphatidylinositol (PtdIns) and PtdIns phosphates as precursors to synthesize phytic acid in leaves (Awad et al., 2012). These myoinositol phosphates play a major role in signal transduction and sugar metabolism for plant growth regulation and seed set. The major enzymes that are manipulated in breeding for lowering phytates are MIPS (myoinositol phosphate synthase), IPK (inositol phosphate kinase), and Mutli-drug Resistant Protein (transmembrane proteins). Genetic manipulation of MIPS was found to decrease phytic acid, resulting in a molar increase in free phosphate. Alteration in the IPK gene reduced phytic acid, accompanied by a limited increase in free phosphate and an increase in the content of lower InsPs. However, alteration of MRP genes lowered phytic acid, resulting in a molar increase in free phosphate in specific seed tissues. Thus, proper strategies have to be adopted to reduce the phytates in crops based on their distribution (Pramitha et al., 2021).

### Raffinose

Raffinose (RF) is a trisaccharide composed of galactose, glucose, and fructose. RFOs is synthesized and stored in monocotyledonous seeds and protects the embryo from maturation. In addition, it acts as a storage metabolite and is observed in the seeds as well as the roots of beans, cabbage, Brussels sprouts, broccoli, and asparagus. Raffinose oligosaccharides (RFOs) act is an oligosaccharide that acts as a stachyose source of energy for seed germination, and its reduction in foods should be carried out in a proper way to substantiate seedling vigor. Furthermore, RFOs acts as a key desiccation protectant in seeds, playing a major role in sugar transport in phloem sap and sugar storage in tubers for active metabolism (Blochl et al. 2008). Hence, RFOs is sustained in plants to regulate storage and transport of sugar in crops and is also produced from a branching pathway of myoinositols that produces phytate. The precursor of raffinose is sucrose, and the key enzymes involved in its synthesis are galactosyl (Gol). *FeGolS* genes have been found to be involved in the synthesis of fagopyritols with the help of UDP-Galacytinol synthase (GolS) and d-chiro-inositol, which are also involved in the production of galactinol that produces raffinose along with sucrose (Tian et al., 2019). RFOs synthesis gene from Falcata medicago namely MfGolS1 enhances freezing and chilling tolerance in transgenic tobacco plants. Hence, RFOs could also be manipulated to enhance cold tolerance in plants (Zhuo et al., 2013).

### Lectins

Lectins are unique among carbohydrates in having the ability to bind sugars. Some of the known lectins in crops include ricin, abrin, and favin. Plant lectins have a major role in host–pathogen interactions, as they have a major role in signaling. In addition, they are known to play a major role in establishing a symbiotic relationship with nitrogen fixers (Kobayashi and Kawagishi, 2014). Lectins are widely present in plants and they vary in their structure across families. They are widely used as antimicrobial, antifungal, and antiviral agents (Mishra et al., 2019). Lectins are predominantly synthesized in plants to selectively bind and detect glycans during a pathogenic infestation (Van Damme et al., 2008). Based on their synthesis in plasma membranes they are classified into G-type, C-type, and L-type lectin receptor kinases (LecRKs). In *Arabidopsis*, the chitin receptor kinases are the major chitin receptors and contain three Lys motifs. Few LecRKs are synthesized during ABA signaling and stomatal immunity (Singh et al., 2012). Tobacco plants express L-type LecRKs, which have a major role in plant immunity, whereas *Medicago* exhibits L-type LecRKs, which are involved in symbiosis (Navarro-Gochicoa et al., 2003; Gilardoni et al., 2011). The functional characterization of *FIBexDB* in flax seeds revealed the predominant role of lectins in cell wall biosynthesis, cytoskeleton functioning, and protein biosynthesis (Petrova and Mokshina, 2022).

### Gossypol

Gossypol is yet another terpenoid observed in cotton seed, stem, flower, and root (Stipanovic et al. 1975). This is a part of the sesquiterpenoid aldehyde pathway, which is highly toxic to humans and offers resistance to various cotton pests, including bollworm (Bottger et al., 1964). During seed germination, the cotyledon acts as a primary site of gossypol accumulation; later gossypol is synthesized in the roots (Meng et al., 1999). δ-Cadinene acts a major precursor to produce different structured enzymes such as methylated hemigossypol, gossypol, hemigossypolone, or heliocides (Cai et al., 2010). Together with (+)-δ-cadinene synthase, P450 is involved in 7-hydroxy-(+)-δ-cadinene for the formation, of enzymes that convert farnesyl diphosphate (FPP) to hemigossypol (Wagner et al., 2015). Thus, gossypol is essential if cotton plants are to withstand bollworm attacks, as it confers host plant resistance.

### Saponins

Triterpenoid saponins are synthesized from an isoprenoid pathway by cyclization of 2,3-oxidosqualene in the mevalonate pathway from acetyl-CoA. This further produces oleanane and its glycosylated forms (SGAs) in the Solanaceae and Liliaceae families. Saponins also act as a phytoalexin during fruit and tuber development in crops (Ribera and Zuñiga, 2012). Phytoalexins are synthesized from the cytosolic mevalonic acid pathway, which produces steroidal glycoalkaloids (SGAs) and cholesterol, which goes through several steps of hydroxylation, oxidation, transamination, and glycosylation (Haralampidis et al., 2002). The isoprenoid mevalonate pathway thus produces cholesterol from acetyl-CoA. Recent studies have revealed that acetate, mevalonate, lanosterol, cycloartenol and deuterium were categorized as cholesterol which are found to be the precursors for SGA in tomatoes (Itkin et al., 2013). Hence, these compounds could be manipulated in crops to confer resistance against diseases in vegetables.

### Goitrogen and Glucosinolates

Another secondary metabolite, known as goitrogen, induces thyroid in tissue and is primarily found in rapeseed, cabbage, and canola seeds. Goitrin (l-5-vinyl-2-thiooxazolidone) is a water-soluble component in plants. Progoitrin is a precursor of goitrin and is produced by the enzyme thioglucosidase from cysteine and methionine (Chandra, 2010). Sufficient genetic variability in the pearl millet germplasm for goitrogens renders the identification and manipulation of genes related to C-C-glycosylfalvones (C-GFs) there by reducing goitrogens accumulation in grains. Glucosinolates are another group of unique secondary metabolites, and are found in the seeds of edible broccoli and plants of the genus *Brassica*. Methionine is also a precursor in the synthesis of glucosinolates, which include allylglucosinolate (sinigrin), glucotropaeolin, gluconasturtin, glucoraphanin, and sulforaphane. These are mainly converted to reactive isothiocyanates in mustard oils, which impart the mustard-like or garlic-like odors associated with horseradish and mustard (Banihani, 2017). Glucosinolates are synthesized from methionine, tryptophan, and seven additional protein amino acids. The pathways of goitrogen and glucosinolates are interconnected, as they are derived from similar precursors through a branched pathway. The breakdown of glycosylates often leads to a bitter taste owing to rancidity (Ishida et al., 2014). Hence, the degradation of amino acids in plants influences the storage quality of the produce.(Boncompagni et al., 2018).

### Tannins

Tannins play a key role in the antioxidant activities of plants and are known to protect crops from pest infestations. They are classified into hydrolyzed tannins and condensed tannins (Khanbabaee and Van Ree, 2001). They are found in fruits such as bananas, blackberries, apples, and grapes. These foods are known to protect humans from cardiovascular diseases, cancer, and osteoporosis. Tannins are also utilized in industry as a natural preservative agent and are reported to possess antibacterial, antiviral, antiparasitic, anti-inflammatory, and anti-diarrheal activity (Tong et al., 2021). The synthesis of tannins takes place in plastids, and they are synthesized from l-phenylalanine *via* the shikimate pathway. The initial step involves the condensation of aldols and is catalyzed by 3-deoxy-d-arabino-heptulosonate-7-phosphate synthase (DAHP), with phosphoenol pyruvate and erythrose-4-phosphate as substrates. The synthesis of tannins in plants is often triggered by mechanical wounding or insect attacks (Mora et al., 2022).

### Oxalic acid

Oxalic acid is a secondary metabolite found in the leaves, fruits, and seeds of *Rumex crispus*, *amaranthus*, *Chenopodium album*, and sugar beet. It is poisonous and can cause headaches, coma, and even death. The oxalic acid metabolic pathway begins with glycine and ends with glyoxylate (Atanassova and Gutzow, 2013). Oxalate is synthesized from three precursors, namely glyoxylate, ascorbate, and oxaloacetate. Their accumulation takes place in the mature leaf lamina and leaf petiole (Cai et al., 2018). The maturing spike transcriptome of finger millet contains major genes of the oxalic acid precursors biosynthesis pathway (*SGAT*, *GGAT*, *ICL*, *GLO*, *MHAR*, *APO*, and *OXO*) (Akbar et al., 2018). Furthermore, it has been observed that these precursors play a major role in climate resilience and growth regulation of crops during pollination (Kobayashi et al., 2014).

### Erucic acid

Erucic acid is a monounsaturated omega-9 fatty acid that is present in the seeds of plants of the genus *Brassica*. It is produced from the anabolic pathway initiating the synthesis of polyunsaturated C_18_ fatty acids *via* desaturation of VLCFAs (very long-chain fatty acids) involving elongation reactions (Venegas-Calerón et al., 2015). Acetyl fatty acid (acetyl-CoA) is synthesized in plastids, and erucic acid is formed from oleic acid by enzymes found in the endoplasmic reticulum. Thus, it is synthesized in the plastid and later exported to the cytosol. The seed lipids with *FAD2* sense overexpression in embryos at mid-maturity exhibit an altered erucic acid content; thus, the *FAD2* gene could be used to alter the erucic acid content of brassicas (Jadhav et al., 2005). Subsequently, Wu et al. (2008) identified that a particular gene, namely the fatty acid elongase 1 gene (*FAE1*), plays a major role in erucic acid synthesis in rapeseed. The sequencing of this gene from a zero erucic acid mutant revealed a four-basepair deletion between T1366 and G1369 that results in a frameshift mutation. This deletion leads to a premature stop of the translation at the 466th amino acid residue. This deletion is predominantly found in the C genome of *Brassica napus*. (Ghanevati and Jaworski, 2001).

### Alkaloids

The quinolizidine alkaloids (QAs) comprise a ring structure and are classified into lupanine, angustifoline, lupinine, sparteine, multiflorine, aphylline, anagyrine, and cytisine. With the exception of anagyrine and cytisine, they are predominantly found in lupins. QAs have bitter taste when consumed and but confer resistance to pests and diseases. The biosynthesis of these alkaloids begins with the decarboxylation of l-lysine to produce cadaverine. This is then followed by oxidative deamination, regulated by copper amine oxidase (CuAO), to yield 5-aminopentanal, and this is further cyclized to Schiff’s base (Frick et al., 2017). The series of reactions after these processes include Schiff’s base formations, aldol-type reactions, hydrolysis, oxidative deamination, and coupling, thereby producing QAs. Until now, only two genes for the biosynthesis of alkaloids have been identified, one of which is *La-L/ODC*, which is a homolog of *ODC*, which is involved in the biosynthesis of a precursor of nicotine biosynthesis. In addition, other genes, namely MIA in *Catharanthus roseus* (vinblastine and vincristine) and BIA in *Coptis japonica* (berberine) and *Papaver somniferum* (morphine) serves as model pathways for identifying candidate genes for genetic manipulation in alkaloids (Bunsupa et al., 2012). Accumulation of alkaloids has also been observed in the aerial tissues and chloroplast in lupins (Frick et al., 2017). Recently, omics techniques have been used to develop low-alkaloid mutants that lead to a reduced alkaloid content in lupins. Gene editing approaches addressing source-to-sink transport in the metabolism of alkaloids are yet to be explored to manipulate alkaloid toxicity (Mancinotti et al., 2022).

## Importance of traditional and processing techniques in overcoming the anti-nutrients in foods

Several traditional processing methods are being followed to enhance the bioavailability of micronutrients in plant-based diets. Today, a variety of methods are employed to counteract the effects of these food anti-nutrients, including milling, soaking, germination, autoclaving, and microwave treatment, as well as fermentation (Samtiya et al., 2020). This section focuses on the processing methods adopted to reduce anti-nutritional traits in crops (**Table 2**). Effective processing techniques adopted for reducing individual anti-nutritional traits are also described. Value-added products made using these techniques have recently become available on the market.

**Table 2:** Effect of different processing techniques to minimize the anti-nutrients in foods.

S. No	Anti-nutrient traits	Traditional methods	Effective method	Reference
1.	Phytic acid	Milling Soaking Germination Fermentation Blanching	Soaking Germination Fermentation	Gupta et al., 2015; Udensi et al., 2008; Greiner and Konietzny, 2006; Coulibaly et al., 2011; Oghbaei and Prakash, 2016; Simwaka et al., 2017
2.	Lectins	Milling Boiling Soaking Fermentation	Soaking Boiling Heating Fermentation	Gupta et al., 2015; Maphosa and Jideani, 2017
3.	Tannins	Milling Soaking Autoclave Germination Fermentation Blanching Boiling	Boiling Soaking	Gupta et al., 2015; Ertaş and Türker, 2014; Patterson et al., 2017; Ogbonna et al., 2012; Simwaka et al., 2017
4.	Saponins	Boiling Washing Fermentation Roasting	Fermentation	Maphosa and Jideani, 2017; Samtiya et al., 2020
5.	Oxalic acid	Milling Blanching Boiling Soaking	Boiling Soaking	Suma and Urooj, 2014; Patel et al.2018
6.	Enzyme inhibitors	Soaking Autoclave Roasting Fermentation Boiling	Fermentation Boiling	Kumari, 2018; Patterson et al., 2017; Vagadia et al., 2017; Ogodo et al., 2019
7.	Polyphenols	Germination Soaking Fermentation	Germination	Singh et al., 2017; Simwaka et al., 2017
8.	Gossypols	Extrusion Fermentation	Extrusion	Buser and Abbas, 2001
9.	Raffinose	De-hulling Germination Alcoholic extraction Microbial treatment	Cooking	Kannan et al., 2018
10.	Goitrogens	Steaming Cooking Fermenting Milling Soaking Washing	Soaking	Bajaj et al., 2016
11.	BOAA	Soaking Boiling Fermentation Cooking Autoclaving	Soaking and cooking	Srivastava et al., 2015; Hailu et al., 2015
12.	Alkaloids	Soaking Washing Germination Fermentation Aqueous thermal treatment Alkaline treatment	Soaking, cooking fermentation, and alkaline treatment	Boschin and Resta, 2013

### Milling

This is the most common technique for separating the bran layer from grains. Since anti-nutritional factors are mostly present in bran, this process removes anti-nutrients and reduces their distribution in grains. This procedure effectively eliminates anti-nutrients in bran, such as phytic acid, lectins, tannins, and enzyme inhibitors (Gupta et al., 2015). A study in pearl millet found that milling altered the chemical makeup and distribution of oxalic acid (Suma and Urooj, 2014). Hence, milling is effective in removing anti-nutrients from aleurone and bran.

### Soaking

Soaking is yet another popular method for removing anti-nutrients from food. Soaking reduces the cooking time and enhances the release of endogenous phytases found in plant foods (Vashishth et al., 2017). Soaking provides essential moist conditions in nuts, grains, and other edible seeds that are required for germination and thereby also reduces trypsin inhibitors and phytic acid to improve digestibility by enhancing the nutritional value of grains (Kumari, 2018). Soaking, boiling and autoclaving was found to be effective to reduce tannins while soaking the seeds for 24 hours drastically reduced the hydrogen cyanide. Further soaking was found to be more helpful in reducing the stachyose and raffinose content with an average reduction of 51.20% and 21.20% respectively (Udensi et al., 2008). Soaking legumes in water overnight has been found to reduce phytate, protease inhibitors, lectins, and tannins. A 12-hour soaking was found to decrease the amount of phytate in peas by up to 9%, while soaking pigeon peas for 6–18 hours reduced the concentration of lectins, tannins, and protease inhibitors by 38–50%, 13–25%, and 30%, respectively (Ertaş and Türker, 2014). It has also been suggested that wheat and barley can be ingested after soaking for a length of time, preferably 12–24 hours (Onwuka, 2006). It has also been reported that soaking grains and beans can successfully enrich the amount of protein and minerals in grains (Coulibaly et al., 2011).

### Boiling

Anti-nutrients such as lectins, tannins, and protease inhibitors can be ameliorated by high heat during boiling. One study found that boiling pigeon peas for 80 minutes reduced protease inhibitors by 70%, lectin by 79%, and tannin by 69% (Onwuka, 2006). It has also been reported that boiling of cooked green leafy vegetables further reduces calcium oxalate by (19–87%) and that boiling is be more efficient than baking and steaming (Amalraj and Pius, 2015). A study by Maphosa and Jideani (2017) found that boiling beans significantly improved their nutritional quality by reducing their lectin and saponin concentrations.

### Autoclaving and Roasting

The majority of foods show health benefits when consumed after autoclaving. The cooking time required depends on the type of anti-nutrient and the cooking method. Generally, the longer the cooking time, the greater the reduction in anti-nutrients. According to earlier research, heating foods significantly increases their nutritional value by removing their content of anti-nutrients, especially tannins and trypsin inhibitors (Patterson et al., 2017). Trypsin inhibitor activity in soybean meal was dramatically reduced by roasting (Vagadia et al., 2017). Another study found that heating, soaking, and autoclaving of beans considerably reduced the amount of enzyme inhibitors and tannins in grains (Torres et al., 2016).

### Sprouting

This is an effective process for lowering the anti-nutrient content in plant-based foods (Nkhata et al., 2018). During sprouting, anti-nutrients such as phytate and protease inhibitors are degraded. Lectins and protease inhibitors have also been found to be slightly reduced. Various kinds of grains and legumes have been enriched by sprouting, which reduced phytate by 37–81%. The enzyme phytase, which is often activated during seed germination, breaks down the phytate–mineral bound complex in grains. Hence, this approach is most usually employed to reduce the anti-nutritional content of cereals (Oghbaei and Prakash, 2016; Vashishth et al., 2017). Azeke et al. (2011) found that the phytate level of cereal grains was considerably lowered after 10 days of sprouting. Recent research also found that activation of beta-glucosidases during germination altered the isoflavone profile of soybeans, and this is significant for boosting nutritional value, as isoflavones have similar chelating effects (Yoshiara et al., 2018; Ida and de Camargo, 2022). In addition, it has been found that, in millets, the greatest reductions in polyphenol concentrations (up to 75%) are obtained by sprouting, exceeding those achieved by soaking, microwave treatment, and fermentation (Singh et al., 2017).

### Fermentation

The metabolic process of fermentation is found to enhance the absorption of nutrients in grains. This also involves the oxidation of carbohydrates to produce energy. Grain nutritional value has been proven to be enhanced by fermentation that involves adding more critical amino acids, including lysine, methionine, and tryptophan (Mohapatra et al., 2019). The crucial process of fermentation dramatically reduces the amount of anti-nutrients such as phytic acid, tannins, and polyphenols in cereals (Simwaka et al., 2017). Tannin levels were found to be reduced by lactic acid fermentation, resulting in increased iron absorption (Ray et al., 2014). In a recent study, using typical fermentation techniques, maize flour was fermented with a mixture of lactic acid bacteria (LAB) for interval periods of 12 hours to examine the impact of fermentation on anti-nutritional components. It was found that anti-nutrients such as tannin, polyphenol, phytate, and trypsin inhibitor were significantly decreased by fermentation and that the reduction in anti-nutrients increased with increasing fermentation time. The results showed that, compared with spontaneous fermentation, anti-nutritional components were lowered more by LAB mixture fermentation (Ogodo et al., 2019).

### Combination of methods

Combining several strategies can significantly reduce anti-nutrients. In some cases, anti-nutrients can be totally eliminated from foods. For example, soaking, sprouting, and lactic acid fermentation reduced phytate in quinoa by 98%. Similarly, sprouting and lactic acid fermentation of corn and sorghum entirely eliminated phytate. Furthermore, soaking and boiling pigeon peas reduced lectins, tannins, and protease inhibitors by 98–100% (Onwuka, 2006). Hence, combining multiple distinct elimination procedures is the most effective way to eliminate anti-nutrients in plant meals.

### Extrusion

In the food industry, extrusion is a widely utilized processing method and has numerous benefits. A single screw or a series of screws are used to push food ingredients through a tiny aperture. It has been found that anti-nutrients such as phytic acid, tannins, phenols, alpha-amylase, and trypsin inhibitors are dramatically reduced by extrusion. Extrusion has also been found to reduce the proportion of phytic acid phosphorus to total phosphorus. Extrusion of legumes that have been previously soaked in water for 16 hours has been recommended to improve their nutritional value, and this has increased their utilization by humans and animals (Abd El-Hady and Habiba, 2003). Tannins in sesame oilseed meal were also reduced using a single-screw frying extruder (Mukhopadhyay and Bandyopadhyay, 2003). Based on the official standard procedures of the American Oil Chemists’ Society, test findings showed that around 71%–78% reduction in free gossypol levels was also effectively attained by extrusion (Buser and Abbas, 2001).

## Breeding strategies to alter the anti-nutritional components for enhanced bioavailability of nutrients in foods

**Figure 1:**
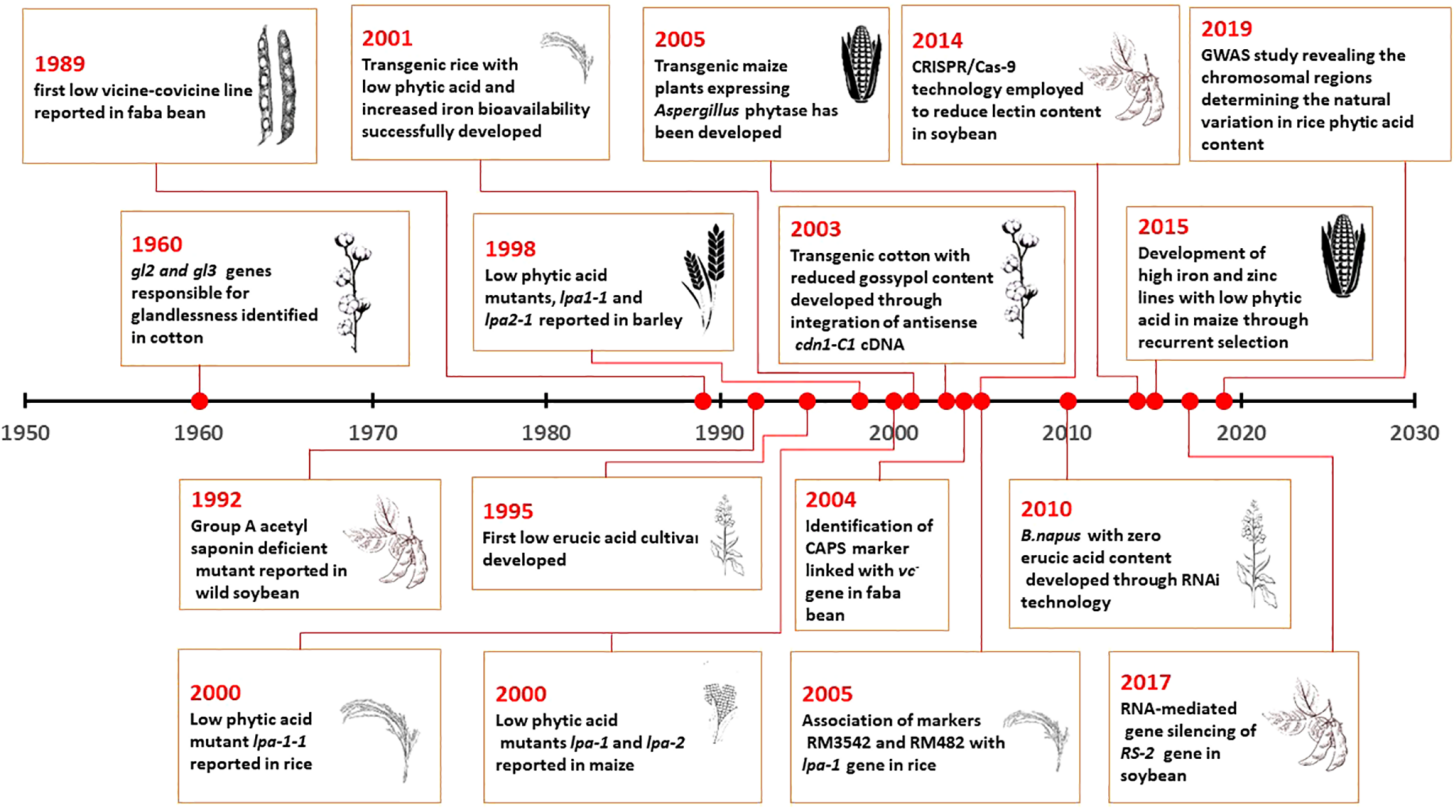
Timeline of Anti-nutritional Breeding in major crops.

The reduction of anti-nutrients in crops is a crucial breeding strategy that plays a major role in enhancing the quality of the produce. Several breeding techniques, starting with selection, mutation, backcrossing, hybridization, and population improvement, have been implied with the natural and induced genetic resources. The breeding for reducing antinutrients in crops was intiated in the early 1960s with glandless cotton (**Figure 1**). More recently, gene silencing and editing techniques have been used to produce low anti-nutrient lines of major crops (**Figure 1**). Conventional breeding for anti-nutrient reduction began with the identification of reduced anti-nutrient accumulation in germplasm accessions. Genotypes with reduced gossypol content were selected in 1960, and McMichael (1960) reported that glandlessness in cotton is conferred by two genes, namely *gl2* and *gl3*. As gossypol plays a major role in host plant resistance, these findings later led to the discovery of an ideal genotype with glandless seed-gossypol cum glanded plant (Dilday, 1986; Vroh Bi et al., 1999). This led to the identification reduced gossypols in seeds without manifesting their concentrations in the vegetative parts.

Subsequently, selection for reduced enzyme inhibitors from pulse germplasm was also observed to be an efficient way to identify potential donors with reduced inhibitors. Zero Kunitz inhibitor lines, namely PI 157-440 and PI 196-168, were identified in soybean (Orf and Hymowitz, 1979). These inhibitors were found to be controlled by a recessive gene, *tj*, which was later introgressed into an elite cultivar by Bernard and Hymowitz in 1986. Similarly, low-vicine and low-covicine lines were selected from the germplasm of 919 accessions in faba bean. The low vicine–covicine trait in pulses was found to be produced by a recessive gene, which was designated “*vc*”, and this was conferred for reducing the enzyme inhibitors (Duc et al., 1989; Duc et al. 2004; Gutiérrez et al., 2006; Webb et al. 2016; O’Sullivan et al. 2018).

Despite these adopted selection techniques, recurrent selection in maize with two synthetic populations, namely BS11 and BS3, was also performed. Three cycles of selection were successful in developing high-iron and high-zinc lines with low phytic acid in maize (Beavers et al., 2015). Similarly, selection for low saponin in quinoa after three cycles of pedigree breeding was found to reduce saponin accumulation in the population, but, due to the dominance of this trait, alternate strategies were required to reduce saponin content in polyploid and heterozygous crops (Ward, 2000). During the course of selection in the same period, there were investigations for induced mutations in the cultivar MACS 450 of soybean by gamma rays. These treatments were able to produce three mutants in M_5_ with lower lectin and normal germination rate. Further, this was also suggested to be used as a potential donor in improvising the soybean meal quality (George et al., 2008).

**Figure 2:**
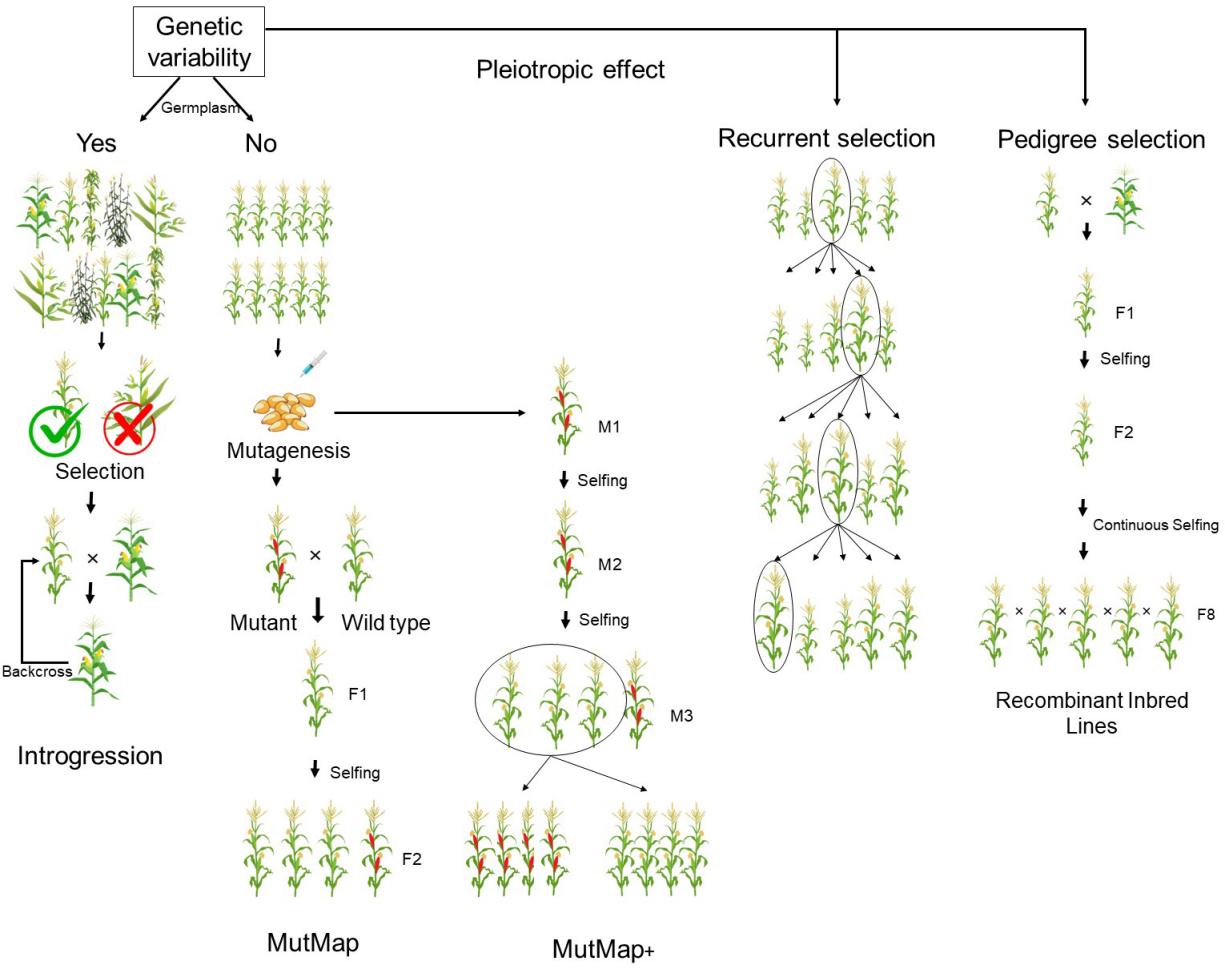
Breeding methods focused for reducing anti-nutritional factors.

Backcrossing and mutation breeding are the strategies predominantly used to reduce anti-nutritional traits in crops (**Figure 2**) (Wilcox et al., 2000; Yuan et al., 2009). This is because several anti-nutritional traits play a major regulatory function in plants (Sureshkumar et al., 2014). Hence, their drastic reduction has been observed to have negative pleiotropic effects, affecting yield (Raboy et al., 2015). For this reason, phytic acid has been successfully reduced in potential donors identified from spontaneous and induced mutants in major crops (Pramitha et al., 2021). Raboy et al. (2000) identified three *lpa* mutants in maize. Among them, the *lpa1* mutant was found to exhibit low phytic acid with meagre accumulation of myoinositol phosphates due to a mutation in the initial biosynthesis of phytic acid involving myoinositol. *lpa2* had reduced phytic acid with accumulation of myoinositol phosphate intermediates, and *lpa3* had reduced phytic acid with accumulation of myoinositol (Shi et al. 2003; Shi et al., 2005). Recent studies have shown that introgression of *lpa 2* in the parents of a ruling hybrid, DMH 121, from the Indian Institute of Maize Research, by marker-assisted backcross was efficient in developing a better version of the released hybrid. The near isogenic lines (NILs) of the parents of DMH 121, namely BML 6 and BML 45, were observed to produce less phytate than the original lines. The newer versions of these parents were exactly the same as the earlier version, except for phytic acid content, and they could be further hybridized to produce low-phytic acid hybrids in maize (Yathish et al., 2022). This coincided with marker-assisted backcross of the null allele for Kunitz trypsin inhibitor (KTI) in DS9712 and DS9814 with a donor called P1542044 in soybean. In this case, in order to minimize linkage drag, three selections, foreground, background, and recombinant, were performed. This resulted in the development of six KTI-free lines in soybean with a maximum recovery percentage (Maranna et al., 2016).

Regarding RFOs, their amount of consumption and the ratio of balanced protein and oil profile in foods are yet to be determined (Elango et al., 2022). Selection for a lower RFOs version of high-RFOs foods such legumes found a negative correlation with protein and yield. In soybean, a significant negative correlation was observed between protein and RFOs, whereas RFOs was reported to have a positive correlation with oil content (Bueno et al., 2018). Studies on RFOs have identified a role for *MIPS* (myoinositol phosphate synthase) and galactinol synthase activity, which was exploited to manipulate RFOs levels in major crops (Elango et al., 2022). Further, genetic mapping for raffinose in recombinant inbred lines (RILs) of soybean produced from a cross of MD96-5722 and Spencer detected 14 major quantitative trait loci (QTLs) for raffinose which could be utilized to produce higher concentrations of sucrose and lower concentrations of raffinose and stachyose in the future (Akond et al., 2015).

Glucosinolates have also been similarly altered by breeding approaches. Glucosinolates were found to have a quantitative inheritance which was highly influenced by environmental factors. A high-density linkage map of the major genes involved in the synthesis of glucosinolates in *Brassica oleracea* has been created with sequences of *BoGSL-ALK*. In addition, comparative genomics studies of the glucosinolate biosynthesis pathway in *Arabidopsis* revealed significant QTLs and candidate genes to alter its profile in crops (Gao et al., 2007; Issa, 2010). This led to the development of high-glucoraphanin broccoli by marker-assisted selection involving an interspecific cross between *B. oleraceae* × *B. villosa*. In addition, marker-assisted selection for altered glucosinolate profiles was achieved between *B. rapa* × *B. oleraceae* (Hirani, 2011). Further projects have focused on developing super broccoli with higher isothiocyanate content by incorporating genes from wild species with the aim of developing pharmaceuticals (Ishida et al., 2014). Similar studies have investigated isothiocyanates and glucosinolates in *Raphanus sativus*, and QTL analyses using high genetic density mapping led to the development of candidate genes for glucosinolate synthesis in roots (Wang et al. 2013). These studies have improved the prospects of altering the profiles of glucosinolates and isothiocyanates (Zou et al., 2013).

Subsequently, breeding to achieve zero erucic acid, due to its serious health issues, was also effective in producing low erucic acid lines in brassicas (Sivaraman et al., 2004). The major gene that plays a role in erucic acid synthesis was observed to be *FAE1*. Sequencing of the *FAE1* gene in high- and low-erucic acid cultivars revealed 28 base deletions containing 24 bases of AT-rich regions in a 1,300-bp section upstream of the promoter of the *FAE1* start codon (Yan et al., 2015). Later, mutations in *FAE1* were induced to identify low erucic acid lines, and introgression of these erucic acid mutant genes in elite cultivars was carried out by Karim et al. (2016). In that study, a genes named *BnFAE1.1* and *BnFAE1.2* in the A and C genome of rapeseed was introgressed to a turnip cultivar. The incorporation of the mutant gene *bnfae1.1(e1)* was monitored by a CAPS (cleaved amplified polymorphic sequence) primer. Early deteriorations in the seed set of backcross progenies were later observed to be improved in the advanced progenies. This suggested that the frequency of recombination events among progenies substantiated the negative effects on morphological traits in later generations (Pramitha et al., 2021).

The overall schemes adopted for marker-assisted breeding and QTLs detected for reducing the anti-nutrients are presented in **Table 3**. It can be seen that, in the earlier reports, anti-nutritional factors were manipulated either by introgression or by mutation breeding. The backcrosses also involved selfing in their intermittent process, as most of the reductions were controlled by recessive genes (**Table 3**). Genetic manipulation of anti-nutrients needs to be carefully monitored, as anti-nutrients play a major role in plant defense and abiotic stress tolerance (Guttieri et al. 2004). Hence, alternate strategies to minimize their negative pleiotropic effects with rapid selection among populations have to be further developed in the future using omics approaches. Upcoming projects involving transgenics and gene editing opens a new gateway to tissue-specific expression, an area that is gaining popularity (Wang et al., 2022).

**Table 3:** Summary of the major QTL’s observed for the anti-nutrients in crops.

S. no.	Crop	Anti-nutrient	QTL/marker	Location	Reference
1	Rice	Phytic acid		Chromosome 2L	Larson et al., 2000
				Chromosome 2	Andaya and Tai, 2005
				Chromosome 5	Stangoulis et al., 2007
				Chromosome 12	
			*qLPA8.1*	Chromosome 8	Gyani et al., 2020
2	Barley	Phytic acid		Chromosome 2H	Larson et al., 1998
3	Barley	Raffinose	*QcRaf.2H*	Chromosome 2H	Gudys et al., 2018
4	Corn	Phytic acid		Chromosome 4	Liu et al., 2013
				Chromosome 6	
				Chromosome 1	
				Chromosome 7	
				Chromosome 2	
5	Mung bean	Phytic acid	*SDPAP4.1*	LG- 4A	Sompong et al., 2012
			*SDPAP11.1*	LG-11A	
6	Pea	Phytic acid		LG-5	Shanmugam et al., 2015
7	Rapeseed	Erucic acid	*qEA.A8.1*	LG-A8	Cao et al., 2010
			*qEA.A8.2*	LG-A8	
8	Indian mustard (*Brassica juncea* L.)	Erucic acid	*ea-1*	LG-17	Gupta et al., 2004
			*ea-2*	LG-3	
			*Eru-A8-1-EJ*	LG-A08	Rout et al., 2018
			*Eru-A8-2-EJ*	LG-A08	
			*Eru-A8-3-EJ*	LG-A08	
			*Eru-A8-1-EPJ*	LG-A08	
			*Eru-A8-2-EPJ*	LG-A08	
			*Eru-A8-3-EPJ*	LG-A08	
			*Eru-A8-1-VH*	LG-A08	
			*Eru-A8-2-VH*	LG-A08	
			*Eru-A8-1-VH*	LG-A08	
			*Eru-B7-1-VH*	LG-B07	
			*Eru-B7-2-VH*	LG-B07	
			*Eru-B7-1-DE*	LG-B07	
			*Eru-B7-2-DE*	LG-B07	
			*Eru-B7-3-DE*	LG-B07	
			*Eru-A8-1-TD*	LG-A08	
			*Eru-A8–2-TD*	LG-A08	
			*Eru-A8-3-TD*	LG-A08	
9	Yellow mustard (*Sinapis alba* L.)	Erucic acid		Chromosome 3	Javidfar and Cheng, 2013
10	Soybean	Gossypol	*qGos1-c13-1*	Chromosome 13	Yu et al., 2012
			*qGos1-c19-1*	Chromosome 19	
			*qGos2-c19-1*	Chromosome 19	
11	Soybean	Raffinose		Chromosome 6	Salari et al., 2021
				Chromosome 6	Skoneczka et al., 2009
			*qRAF001*	Chromosome 1	Akond et al., 2015
			*qRAF002*	Chromosome 3	
			*qRAF003*	Chromosome 6	
			*qRAF004*	Chromosome 9	
			*qRAF005*	Chromosome 14	
			*qRAF006*	Chromosome 14	
			*qRAF007*	Chromosome 16	
12	Soybean	Stachyose	*qSTA001*	Chromosome 1	Akond et al., 2015
			*qSTA002*	Chromosome 6	
			*qSTA003*	Chromosome 12	
			*qSTA004*	Chromosome 14	
13	Soybean	Group A saponin		Chromosome 15	Sundaramoorthy et al., 2018
		Group A saponin (hypocotyl)		Chromosome 5 (A1)	Teraishi et al., 2017
				Chromosome 8 (A2)	
		Group A saponin (cotyledon)		Chromosome 6 (C2)	
14	Faba bean	Vicine–convicine		Chromosome 1	Khazaei et al., 2015
15	Sorghum	HCN (Dhurrin)	*Dhu1*	Chromosome 1	Hayes et al., 2016
		Hydrocyanic acid	*qPA7-1*	Chromosome 4	Wu et al., 2022
16	Sorghum	Tannin	*Qsqr.t-2*	Chromosome 4	Wu et al., 2012
			*Qsqr.t-4*	Chromosome 4	
17	Field mustard (*Brassica rapa*)	Glucosinolates		LG-A3	Hirani, 2011
18	Rapeseed (*Brassica napus* L.)	Glucosinolates	*GSL-1*	LG-20	Toroser et al., 1995
			*GSL-2*	LG-1	
19	Indian mustard (*Brassica juncea* L.)	3-Butenyl-glucosinolates	*GSL-A2a*	LG-2a (A)	Mahmood et al., 2003
			*GSL-A2b*	LG-2b(A)	
		2-Propenyl-glucosinolates	*GSL-A2a*	LG-2a (A)	
			*GSL-F*	unlinked segment	
			*GSL-B3*	LG-3 (B)	
20	*Barbarea vulgaris*	NAS, 2-phenylethylglucosinolate (gluconasturtiin)	*qNAS-4-1*	LG-4	Liu et al., 2019
			*qNAS-4–2*	LG-4	
		BAR, (2*S*)-2-hydroxy-2-Phenylethylglucosinolate (glucobarbarin)	*qBAR-3-1*	LG-3	
			*qBAR-4-1*	LG-4	
			*qBAR-5-1*	LG-5	
		EBAR, (2*R*)2-hydroxy-2-phenylethylglucosinolate (epiglucobarbarin)	*qEBAR-3–1*	LG-3	
			*qEBAR-4-1*	LG-4	
			*qEBAR-5-1*	LG-5	
		IM, 3-indolylmethylglucosinolate (glucobrassicin)	*qIM-4-1*	LG-4	
			*qIM-6-1*	LG-6	
		4mIM, 4-methoxy-3-indolylmethylglucosinolate (4-methoxyglucobrassicin)	*q4mIM-4-1*	LG-4	
			*q4mIM-5-1*	LG-5	
21	Narrow leaf lupin (*Lupinus angustifolius*)	Quinolizidine alkaloids	*iuc_RAP2-7-pauper loci*	LG-07	Kroc et al., 2019
22	White lupin (*Lupinus albus* L.)	Quinolizidine alkaloids	11 loci	LG-11	Phan et al., 2007
			Pauper loci	LG-18	Rychel and Książkiewicz, 2019
23	Yellow lupin (*Lupinus luteus* L.)	Quinolizidine alkaloids	*YL-06 loci*	LG-06	Iqbal et al., 2020

## Advanced genetic approaches for developing sustainable food crops in future

Manipulating anti-nutritional traits to enhance the bioavailability of nutrients is a major concern in crops, as these traits have to be mitigated in such a way as to avoid negative influences on yield. The reduction of these traits in crops has been successfully carried out for major anti-nutrients and the various methods of altering their content are described in **Table 4**. Gene silencing using RNAi technology is an efficient way of optimizing the expression of these factors in crops and has been applied to the genes involved in the biosynthesis of these components in plants. Gossypol is one plant phytochemical that plays a major role in host plant resistance and is not needed in human nutrition. Therefore, ultra-low-gossypol cotton has been developed by silencing of *δ-cadinene synthase* gene. The knockdown of this gene reduced the accumulation of gossypol in seeds, foliage, and floral organs of transgenic cotton. The initial version of transgenic cotton showed on-par performance, in terms of yield and fiber quality, with stable expression. The transgenic cotton was also observed to exhibit a higher oil content than the control (Palle et al., 2013). Recently, selective RNAi knockout of the *δ-cadinene gossypol* gene in seeds of the cultivar TAM66274 effectively reduced the oil content by about 97%, and the cultivar also passed food safety tests conducted by the Food and Agricultural Organization of the USA (FAO) (Rathore et al., 2020). This method has also been found to be effective in controlling gossypol levels in seeds without affecting gossypol concentration in the vegetative parts, and the technique has been patented by Texas A & M university. Thus, transgenic cotton would contain either a *δ-cadinene synthase* gene or a *δ-cadinene-8 hydroxylase* gene, or both, linked to a seed-specific promoter gene for inducing RNA gene silencing when expressed in cottonseed of the plant (Rathore et al., 2009).

**Table 4:** Major RNAi and gene editing techniques adopted in major crops.

S. no.	Anti-nutrient	Crop	Gene/enzyme	Pathway	Technique	Reference
1.	Phytic acid	Rice	*IPK1*	Phosphorylation of Ins(3)P and phospholipase C mediated	Chromosome mapping	Ye et al., 2013
			*lpa-1*	Lipid independent	Gene editing	Watanabe et al., 2018
			*OsITP5/6K/1*	Lipid dependent phytic acid biosynthesis Lipid independent phytic acid biosynthesis pathway	RNAi-mediated down-regulation	Karmakar et al., 2020
			*OsIPK1*	Inositol phosphate pathway	RNAi-mediated seed-specific silencing	Ali et al., 2013
			*ITPK, OsITP5*	Myoinositol pathway	RNAi-mediated down-regulation	Karmakar et al., 2020
			*RINO1*	Myoinositol pathway and direct proanthocyanidin pathway	Antisense cDNA approach	Kuwano et al., 2009
		Wheat	*TaIPK1*	Auxin signaling pathway	RNAi technology	Ibrahim et al., 2022
			*TaABCC13*	Myoinositol pathway	RNAi technology	Bhati et al., 2016
		Soybean	*GmMIPS-1*	Lipid dependent/salvage pathway	Gene silencing	Nunes et al., 2006; Kumar et al., 2019
			*GmIPK1*	Both lipid independent and lipid dependent	CRISPR/Cas-9 genome editing	Song et al., 2022
			*lpa1*	Myoinositol hexa-kis phosphate	EMS approach	Gillman et al., 2011
			*Glyma.20G085100*	–	QTL mapping	Marsh et al., 2022; Jha et al., 2022
		Barley	*lpa-1-1*	Signaling pathway	QTL mapping	Bregitzer and Raboy, 2006
		Corn	*ZmMRP4* in *lpa-2*	Supply pathway	C-T transition by mutation	Tamilkumar et al., 2014
			*ZmIPK1*	Inositol phosphate pathway	ZFN approach	Shukla et al., 2009
			*lpa2–1*	Myoinositol InsP_6_ pathway	EMS Mu insertion approach	Raboy et al., 2000
			*2.lpa1–1*	Myoinositol phosphate pathway	Gene silencing	Shi et al., 2007
		Rapeseed	*BnITPK*	Both lipid dependent and lipid independent	CRISPR/Cas-9 gene editing	Sashidhar et al., 2020
		Arabidopsis	*AtIpk1-1*	–	T-DNA insertion method	Stevenson-Paulik et al., 2005
			*AtITPK1, AtITPK4*	Inositol InsP_6_ pathway	Reverse genetic approach	Kim and Tai, 2011
			*atips1, atips2*	–	T-DNA insertion method	Kim and Tai, 2011
		Lablab bean	*dlMIPS*	Myoinositol phosphate synthase	RT-PCR system	Jagal Kishore et al., 2020
2.	Erucic acid	Rapeseed	*FAE1*	–	Gene editing	James et al., 1995; Yan et al., 2015
			*FAD2* and *FAE1*		Gene silencing	Peng et al., 2010
			*BnFAE1.1*	Ketoacyl-CoA synthase	RNAi silencing	Tian et al., 2011; Kaur, 2018
			*BnFAE1*		RNAi silencing	Shi et al., 2015
			*BnFAE1* and *BnFAD2*	Long-chain fatty acid biosynthesis	CRISPR/Cas9-mediated gene editing	Shi et al., 2022
			*BnFAD2* and *BnFAE1*	Fatty acid biosynthesis	RNAi silencing	Shi et al., 2017
		Indian mustard	***BjFAE1***	Ketoacyl-CoA synthase	*Agrobacterium*-mediated transgenic method	Kanrar et al., 2006
		Ethiopian mustard	*FAD2* and *FAE*	Fatty acid biosynthesis	Hairpin-RNA mediated silencing	Mietkiewska et al., 2008
3.	Gossypol	Cotton	*Cad1-A*	d-Cadinene synthase	RNAi technology	Davis et al., 1996; Luo et al., 2001
			*gl_2_* and *gl_3_*	d-Cadinene synthase	Southern analysis	Martin et al., 2003; Benedict et al., 2004; Sunilkumar et al., 2006
			*gl_1_, gl_2_*, and *gl_3_*	d-Cadinene synthase	RNAi silencing	Palle et al., 2013; Rathore et al., 2020
			*GhMYB25*	–	Antisense gene silencing	Abdurakhmonov et al., 2016
			*GhCLA1*	–	Temperature-sensitivity CRISPR/LbCpf1-mediated genome editing	Li et al., 2021
			*GhCLA1*	–	CRISPR/Cas-9 technology	Wang et al., 2018
			*CYP82D109*	Gossypol biosynthesis pathway	RNAi technology	Wagner et al., 2015
4.	Lectin	Soybean	*P34* allergen	–	CRISPR/Cas-9 technology	Watanabe et al., 2018
		Peanut	*Gly1 protein*	NAD-dependent	2-D gel electrophoresis	Kottapalli et al., 2008
5.	Saponin	Soybean	*GmBAS1, GmBAS2*	β-Amyrin synthase	RNAi-mediated gene silencing	Takagi et al., 2011
			*DeF26G1*	Flavonoid biosynthesis	Transcriptome profiling	Kuma A, et al., 2016
		Barrel medic	*CYP93E2*	–	*Agrobacterium*-mediated transformation	Confalonieri et al., 2021
		Korean ginseng	*CYP716A53v2*	PPT synthase	CRISPR/Cas9-mediated gene knockout	Choi et al., 2022
			*CYP716A53v2*	PPT synthase	RNAi technology	Park et al., 2016
6.	Tannin	Quaking aspen	*MYB134*	CT biosynthesis	RNAi suppression	Gourlay et al., 2020
		Peanut	*aflS/aflJ*, *aflR*, *aflC/pksA/pksL1*, *pes1*, *afelp*	–	RNAi silencing	Arias et al., 2015
7.	Oxalic acid	Soybean	*b-ODAP*	–	Transgenic production	Kumar V, et al., 2016
		Wheat	*BoGSL-ELONG, BoGSL-PRO*, and *BoGSL-ALK*	Glucosinolate biosynthesis pathway	Comparative genomic analysis (QTL mapping)	Ishida et al., 2014
		Tomato	*FvOXDC*	Oxalic acid biosynthesis pathway	Metabolic remodeling	Chakraborty et al., 2013
		Tobacco	*Germin gf-2.8*	Co-A-dependent pathway, jasmonate pathways, and phenylpropanoid pathways	Transgenic approach	Kumar et al., 2019
8.	Vicine and convicine	Faba bean	*vc, vcr*	–	QTL mapping	Khazaei et al., 2019
9.	Enzyme inhibitors	Finger millet	*Opaque_2_*	–	Random amplified polymorphic DNA (RAPD) and simple sequence repeat (SSR) profiling	Vinoth and Ravindhran, 2017
		Durum wheat	*0.28 ATI*	ATI pathway	CRISPR/Cas-9 multiplex editing	Camerlengo et al., 2020
		Bread wheat	*CM3, CM16 and 0.28 ATI*	ATI pathway	RNAi silencing	Kalunke et al., 2020
10.	Glucosinolate	Wild cabbage	*BjMYB28*	Aliphatic glucosinolate biosynthesis	RNAi targeted suppression	Augustine et al., 2013
		Chinese kale	*BoaMYB28*	Aliphatic glucosinolate biosynthesis	RNAi approach	Yin et al., 2017
		Indian Mustard	*BjuMYB28*	Aliphatic glucosinolate biosynthesis	Intron-spliced hairpin RNAi targeting	Augustine and Bisht, 2019
			*BjuXLG*	Aliphatic glucosinolate biosynthesis	RNAi based suppression	Tiwari et al., 2021
		Rapeseed	*MAM*	Aliphatic glucosinolate biosynthesis pathway	RNAi silencing	Liu et al., 2011
			*BrGI*	GSL biosynthetic pathway	RNAi knockdown	Kim et al., 2021
		Arabidopsis	*HAG1/MYB28*	Aliphatic glucosinolate biosynthesis pathway	RNAi knockdown	Gigolashvili et al., 2007
			*OBP2*	IAA biosynthetic pathway	RNAi mediated	Skirycz et al., 2006
		Garden cress	*LcIND*	Glucosinolate biosynthesis pathway	RNAi mediated	Karmakar et al., 2020
11.	Alkaloids	Potato and Tomato	*Steroidal glycoalkaloids*	Cytosolic mevalonic acid pathway	Silencing glycoalkaloid metabolism 4	Itkin et al., 2013
			*Steroidal glycoalkaloids (SSR2)*	Cytosolic mevalonate pathway	Gene silencing	Cárdenas et al., 2015
		Tobacco and *Catharanthus roeus*	*Steroidal glycoalkaloids (SSR2)*	Mevalonate pathway	Gene editing	Cárdenas et al., 2016
12.	HCN	Cassava	*MeCYP79D1*	Cyanogenic glycoside biosynthetic pathway	CRISPR/Cas9-mediated genome editing	Juma et al., 2022
			*CYP79D1* and *CYO79D2*	Cyanogenic glycoside biosynthetic pathway	CRISPR/Cas9-mediated knockout	Gomez et al., 2021
		Sorghum	*CYP79A1*	Cyanogenic glycoside pathway	Antisense approach	Pandey et al., 2019
13.	BOAA	Grass pea	*β-ODAP*	β-ODAP biosynthesis pathway	CRISPR/Cas9-mediated gene editing	Das et al., 2021

Metabolite engineering for manipulating the concentration of raffinose in soybean was carried out by Valentine et al. (2017). For reducing the concentration of raffinose, the *raffinose synthase 2* gene (*RS2*) was down-regulated by an RNAi construct. The silencing of this gene was further confirmed by qPCR and the total metabolizable energy for soybean meal in poultry was increased from 2,411 kcal/kg to 2,703 kcal/kg in the transgenic soybean. In contrast to this approach, the suppression of the *cucumber stachyose synthase* gene (*CsSTS*) by RNAi-mediated silencing had a significant impact on phloem loading, carbohydrate metabolism, and low-temperature stress tolerance (Lü et al., 2017). Recently, an advanced technique involving gene editing with two guide RNAs to knock out *GmGoLS1A* and *GmGoLS1B* (galactinol synthase genes) resulted in a reduction of raffinose from 64.70 mg/g to 41.95 mg/g (a 35% decrease) in soybean. The developed lines from these knockouts established a higher verbascose, protein, and fat content with no effect on plant growth, suggesting that they are potential targets for altering raffinose in soyabean genotypes (Le et al., 2020).

Adding to these findings, RNAi-mediated silencing of three amylase and trypsin inhibitor genes, namely *CM3, CM16*, and *O.28* (α-amylase/trypsin inhibitors genes), revealed a higher trypsin inhibition which was acceptable to non-celiac wheat-allergic patients. Although there were some alterations in inhibitors, there were no changes in in the high-molecular-weight glutenin subunits or in yield (Kalunke et al., 2020). However, trypsin inhibitors such as TcTI from cocoa provide significant defense against *Helicoverpa* (do Amaral et al., 2022), and trypsin inhibitors that hinder digestion were also recently reported to be effective biopesticides (Rodríguez-Sifuentes et al., 2020). Advanced gene editing techniques targeting two seed-specific KTI genes, namely *KTI1* and *KTI3*, resulting in small deletions and insertions in soybean open reading frames, offer an alternate strategy, by focusing on reducing trypsin inhibitors only in seeds for consumption, will be helpful in the future (Wang et al., 2022).

In addition to the above anti-nutrients, saponin has also been modified by RNAi-mediated silencing of two β-amyrin synthase genes (*GmBAS1* and *GmBAS2*), and has a seed-specific promoter involved in the production of β-conglycinin, a seed storage protein in soybean (Takagi et al., 2011). Subsequently, metabolite remodeling of oxalate-by-oxalate decarboxylase (OXDC) effected a 90% reduction in oxalate, accompanied by with higher calcium, iron, and citrate, in transgenic tomatoes. Proteomic analysis of the *OXDC* leading to concerns that manipulation of this gene would also have undesirable effects unless tissue specific expression can be achieved (Chakraborty et al., 2013). Similarly, the use of RNAi to alter *MYB134* to reduce tannins in poplar plants resulted in enhancing the susceptibility of the plant toward oxidative stress, emphasizing the importance of tissue-specific expression when reducing anti-nutrients in crops (Gourlay et al., 2020). Several approaches, including targeted silencing of *IPK1* genes for lowering phytic acid in rice seeds (Ali et al., 2013), *BjuMYB28* to reduce glucosinolates in brassicas (Augustine et al., 2013), *OXDC* in grass pea and soybean (Kumar V, et al., 2016), *ITPK* genes for reducing phytate in rice and wheat seeds with increased iron and zinc (Lucca et al. 2001) (Aggarwal et al. 2018; Pandey et al. 2021; Karmakar et al., 2020), have been successful in reducing anti-nutrients with minimum effects on morphological performance. Following the success of RNAi in *IPK* to reduce phytic acid, the CRISPR-Cas9 method has recently been used on a similar gene in soyabean, named *GmIPK*, to alter the phytate concentrations in soybean. This experiment was intended to standardize stable transformation of transgenic soybean lines with edited *GmIPK2.* This further emphasized the focus on implying more bioinformatic tools and study on transient expression which are necessary in future to further to improvise the soybean meal quality by CRISPR (Jose et al., 2022).

**Figure 3:**
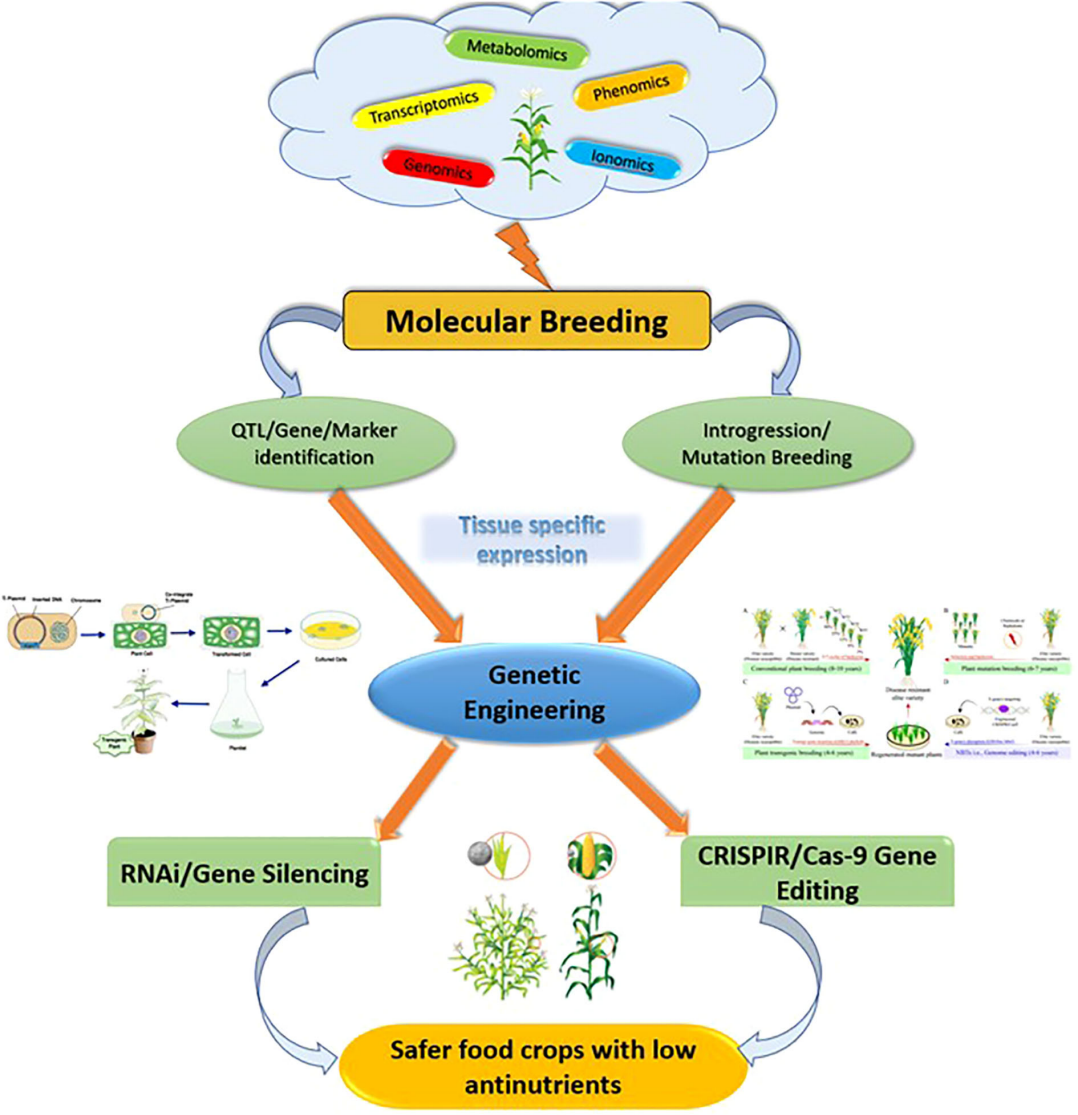
Future prospects of improving the quality of food crops.

Considering the earlier observations for reducing the anti-nutrients, it can be observed that RNAi and gene editing are the two major techniques that are used in tissue-specific reduction in major crops (**Figure 3**) (Perera et al. 2018; Elkonin et al. 2021). Although the initial investigations have been conducted with reduced anti-nutrients, a standard protocol for strategic reduction of anti-nutrients is crucial in crops such as pulses needs to be reinforced in future. Legume-based foods are often reported to hinder the digestion process and, thus, standardization of protocols for seed-specific expression establishes a pathway to a sustainable diet in near future (Drakakaki et al. 2005). In addition, integrative omics will play an important role in the development of low-anti-nutrient versions of other major food crops and for detecting low anti-nutrient donors (Parca et al., 2018; Pandey et al. 2021).

## Conclusion

Reducing anti-nutritional traits in crops is essential factor to achieve higher mineral bioavailability in foods. Although anti-nutrients pose a serious threat to human health, owing to their toxicity, some of them, such as phytic acid, raffinose, tannins and gossypol, are beneficial to growth and metabolism in plants. These anti-nutrients have both favorable and undesirable properties. On the one hand, they favor plant growth through regulatory activities such as biotic and abiotic stress tolerance. On the other hand, they hinder mineral absorption. This restrains any approach that focuses on a threshold reduction in anti-nutritional traits in major food crops. Despite this, a few anti-nutritional factors, such as Kunitz inhibitors, glucosinolates, tannins, alkaloids, and saponins, are being employed in the biopesticides and pharmaceutical industries. Therefore, a constitutive focus on manipulating this content for specific purposes needs to be ensured in future. This would facilitate safe consumption and processing of foods for the upcoming generation for specific anti-nutrients individually to avoid food allergies in future. Several techniques have been employed to alter the accumulation of anti-nutrients in grains, but the use of advanced omics techniques in genomics-assisted breeding, in the case of the majority of anti-nutrients, remain unused. Hence, omics offer a new gateway to understanding the regulatory pathways of crucial anti-nutritional traits in plants and their genetic manipulation. Recently, the use of mutation breeding, introgression, RNAi technology, and gene editing by CRISPR/Cas9 have enable us to achieve seed-specific expression in crops. Thereby, anti-nutrients that confer regulation of vegetative growth and their activity will remain unaffected. To conclude, we could observe that the expression of these anti-nutritional factors varies from crop to crop and, based on their intake, a specific strategy has to be adopted in major crops to provide high-value nutritional foods in future.

## Author contributions

All authors listed have made a substantial, direct, and intellectual contribution to the work, and approved it for publication.
